# Gain Enhancement of a Multiband Resonator Using Defected Ground Surface on Epoxy Woven Glass Material

**DOI:** 10.1155/2014/159468

**Published:** 2014-04-27

**Authors:** Md. Shahidul Alam, Mohammad Tariqul Islam, Haslina Arshad

**Affiliations:** ^1^Department of Electrical, Electronic and Systems Engineering, Faculty of Engineering and Built Environment, Universiti Kebangsaan Malaysia (UKM), 43600 Bangi, Selangor, Malaysia; ^2^Centre of Artificial Intelligence Technology, Faculty of Information Science and Technology, Universiti Kebangsaan Malaysia (UKM), 43600 Bangi, Selangor, Malaysia

## Abstract

A multiband microstrip resonator is proposed in this study which is realized through a rectangular radiator with embedded symmetrical rectangular slots in it and a defected ground surface. The study is presented with detailed parametric analyses to understand the effect of various design parameters. The design and analyses are performed using the FIT based full-wave electromagnetic simulator CST microwave studio suite. With selected parameter values, the resonator showed a peak gain of 5.85 dBi at 5.2 GHz, 6.2 dBi at 8.3 GHz, 3.9 dBi at 9.5 GHz, 5.9 dBi at 12.2 GHz, and 4.7 dBi at 14.6 GHz. Meanwhile, the main lobe magnitude and the 3 dB angular beam width are 6.2 dBi and 86°, 5.9 dBi and 53.7°, 8.5 dBi and 43.9°, 8.6 dBi and 42.1°, and 4.7 dBi and 30.1°, respectively, at the resonant frequencies. The overall resonator has a compact dimension of 0.52*λ*  × 0.52*λ*  × 0.027*λ* at the lower resonant frequency. For practical validation, a lab prototype was built on a 1.6 mm thick epoxide woven glass fabric dielectric material which is measured using a vector network analyzer and within an anechoic chamber. The comparison between the simulated and measured results showed a very good understanding, which implies the practical suitability of the proposed multiband resonator design.

## 1. Introduction

The microstrip resonators are popularly used in versatile applications starting from the mobile radio and wireless communications to the biomedical applications, aircraft, spacecraft, remote sensing, radars, satellites, and space programs. This type of resonators is very demanding due to their merits like light weight, low fabrication cost, robustness, ease of mounting on the host surface, and integration with printed circuits [1]. Microstrip resonators, however, have some drawbacks, for example, narrow bandwidth, low gain, poor radiation, and relatively larger size. To overcome these drawbacks various research initiatives are found in the literature [2–4]. An initial approach is the selection of thick-low permittivity dielectric (substrate) material and electromagnetic coupled feeding method, which usually provides wider bandwidth at higher cost and introduces fabrication difficulty. However, increasing dielectric material thickness will launch stronger surface waves [5, 6]. The surface waves can be suppressed by lowering the effective dielectric constant using micromachining techniques, synthesized low permittivity material, or artificial engineered structures like electromagnetic band gap (EBG) materials or defected ground planes [6, 7]. Also, a wideband miniature design can be achieved by modifying the main radiator to different shapes and incorporating various slots and slits onto it [8, 9].

Besides the need for a broadband resonator, multiband resonators are also very demanding to support multioperation with a single resonator element. A resonator can be used for receiving and transmitting signals at multiple frequencies, which will lead to a compact system design by replacing required several resonators with only one resonator. Moreover, multiband resonators have the capability of reducing the required intermodulation frequency term filters (IMTF) [10]. Resonators have been investigated in different ways to obtain multiband operation such as concentric arrangement of multiple squared split ring microstrip resonators and fed by an L-probe [11]; resonator design consists of two substrates with EMC feeding technique [12], integration of a right isosceles triangular radiator with a pair of monopoles, a semicircular radiator integrated with a slot and a monopole [13], combination of a rectangular ring microstrip and a folded meandered arm [14], and a two-element array of aperture-coupled equilateral triangular microstrip resonator [15]. Even so, these resonator configurations are not structurally simple and require combination of different radiator shapes that increases the chance of performance degradation.

Recently, artificially modified structures such as artificial magnetic ground (AMG) and defected ground surface (DGS) are being investigated as ground plane in designing compact and high performance resonator [5, 16–19]. The defected structures have also been found advantageous in designing microwave filters, transmission lines, power amplifiers, dividers, and combiners [20–23]. A DGS can be realized by etching off some simple shaped portion of the ground plane in a regular or irregular manner. Depending on the defect's shape and dimensions, the shielded current distribution in the ground plane is disturbed, which helps to obtain unwanted frequency rejection. It also changes the transmission line characteristics, for example, capacitance and inductance, resulting in a controlled excitation and propagation of the electromagnetic waves through the dielectric substrate layer [16].

In [24] a spiral-shaped defected ground structure (DGS) was combined with an inset feed microstrip resonator and thus obtained a gain of about 4 dB. Each of the DGS cells was comprised of four spiral arms. In [17] dumbbell shaped DGS was implemented for efficiency enhancement, and in [18] circular slot DGS was applied to suppress resonators cross polarization level. Again, a hexagonal DGS was used to enhance radiation properties of a triangular microstrip resonator array in [19]. Eventually, a DGS enhances resonator performances without any modification of resonator shape and size except the ground plane. However, the above discussed works on defected ground microstrip resonator were concerned with enhancing single band resonator performance rather than any multiband resonator design. Therefore, this work intended to study a multiband resonator with a DGS and investigate the performance enhancement due to the defected ground in comparison to a normal ground plane. We have designed a multiband microstrip resonator incorporating rectangular radiator with embedded rectangular slots. The conventional plain ground is replaced by a DGS which helps to further improve the resonator performances. The design, analyses, and comparisons of the resonator performances are described through a wide parametric investigation in the following sections.

## 2. Design and Geometry of the Symmetrical Slot Multiband Resonator

**Figure 1:**
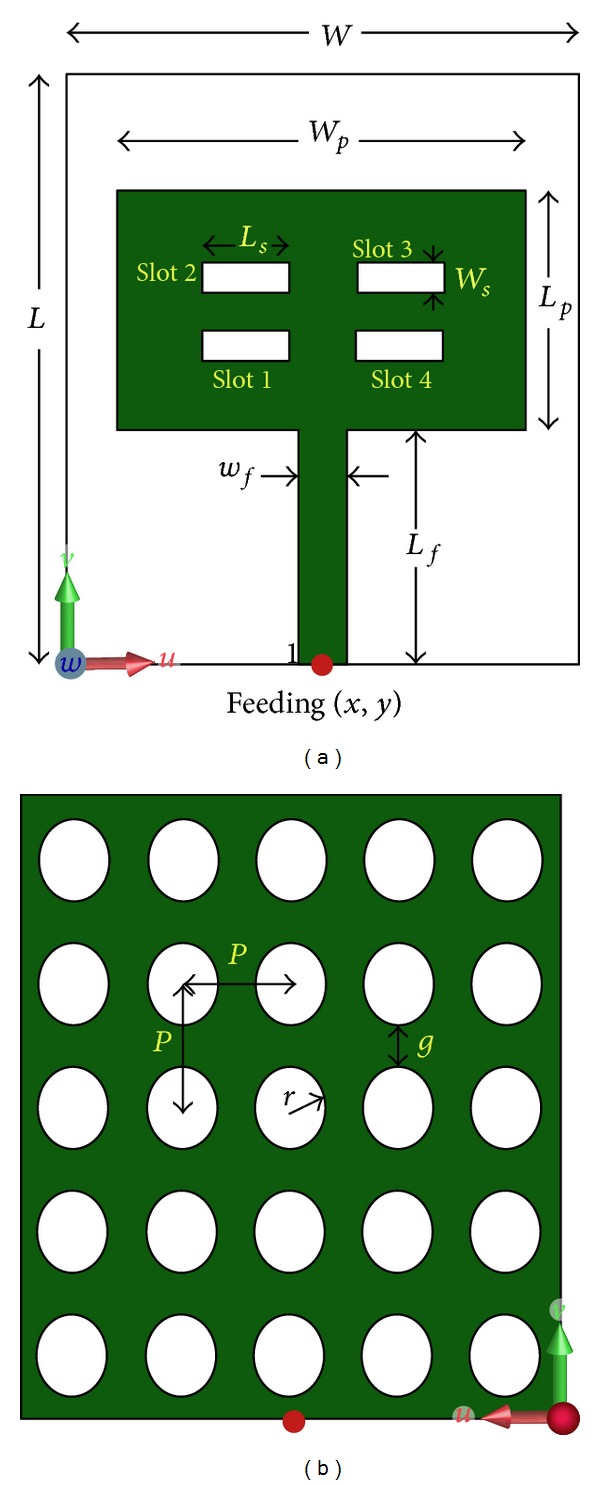
Geometry of the proposed multiband resonator, (a) front and (b) back view.

The schematic diagram of the proposed multiband resonator is depicted in Figure 1. The resonator configuration consists of a rectangular radiator with four rectangular slots which are symmetrical along the feed line. It is fed by a 50 *Ω* microstrip line and built on a double-sided 1.6 mm thick dielectric material, which has a dielectric constant of 4.5 and a tangent loss of 0.02. It is based on epoxide woven glass fabric laminated sheet of flame retardant, grade 4, which has copper cladding of 35 micron (1 oz) on both sides. This material is very cost effective, compatible with a large number of electronic applications and meets most consumer application needs. Thus it is chosen for the proposed resonator design to cope with the most of the applications.

Starting with a microstrip line fed simple rectangular radiator and successively introducing the rectangular slots, the final configuration is obtained. At the very beginning the resonator was designed with a conventional ground plane, which is replaced by a DGS later on, as shown in Figure 1(b). The proposed defected ground is realized by etching out circular areas at a regular distance/periodicity. The etched circular defect has a radius of *r*, the edge to edge separation between two defects is *g*, and the periodicity is *P*. The finite integration technique (FIT) based electromagnetic simulator CST microwave studio is utilized for the analysis.

## 3. Electromagnetic Performance Analysis

The multiband resonator has been studied with several parametric analyses. In order to examine the effects of several variable design parameters, the parametric investigations are conducted and are detailed in the subsequent sections. After the selection of the feeding location (*x*, *y*), the effects of feed line width (*wf*), various slot arrangements, slot length (*Ls*) and slot width (*Ws*), and so forth were examined consequently. Furthermore, the effect of circular defects is also observed by varying defect radius *r* and gap *g*.

### 3.1. Selection of the Feed Position (*x*, *y*) and Feed Line Width (*wf*)

**Figure 2:**
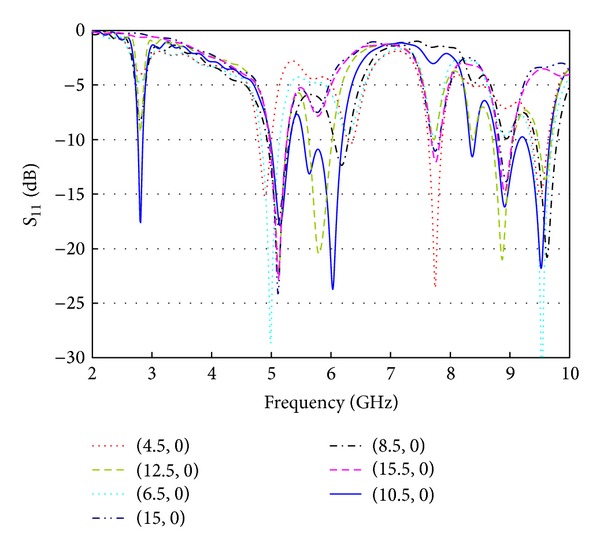
Selection of the feeding position (*x*, *y*).

At the very first, the return loss characteristics (*S*_11_) are computed for different feeding locations and the variations are shown in Figure 2. The importance of feed point selection is quite obvious from the observation of resonance nature with respect to the excitation point. Depending on the feed location the resonator can operate from dual band to hexaband mode. The feed position shifting is considered at 0.5 mm steps; however, the return loss curves are plotted for a 2 mm step to make a clearly traceable view. It can be noticed that when the resonator is fed at the center of the radiator, it shows three resonances, but while the feed point is offset from the center, more resonances are occurring. Similar things happen when the feed point is gradually moving from the radiator edge to the radiator center. Thus it would be better to choose a feed point in between the radiator center and the radiator edge. With respect to the feeding position, the input impedance characteristics are varied, and hence the resonating point, minimum return loss level, and the impedance bandwidth are also affected.

**Figure 3:**
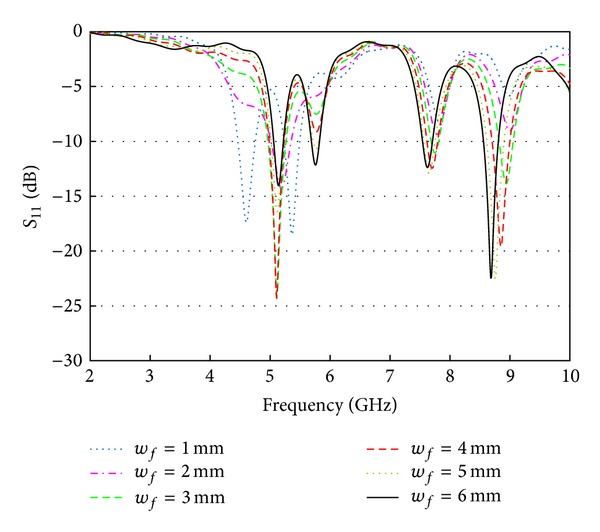
Effects of feed line width (*wf*).

After this, the effects of the feed line width (*wf*) on the resonator behavior are also observed.  Figure 3 demonstrates the return loss variations with respect to the *wf* values of the proposed resonator. The first resonance is very sensitive to the width (*wf*), whereas the other resonances are less sensitive to it in the way that they almost retained their positions but the return loss level. The feed line width affects the impedance nature; thus the resonances are shifted, especially the fundamental resonance. Despite of the variation of the feed line width, still the resonator preserves the multiband characteristics.

### 3.2. Effects of Slot Width (*Ws*) and Slot Length (*Ls*)

**Figure 4:**
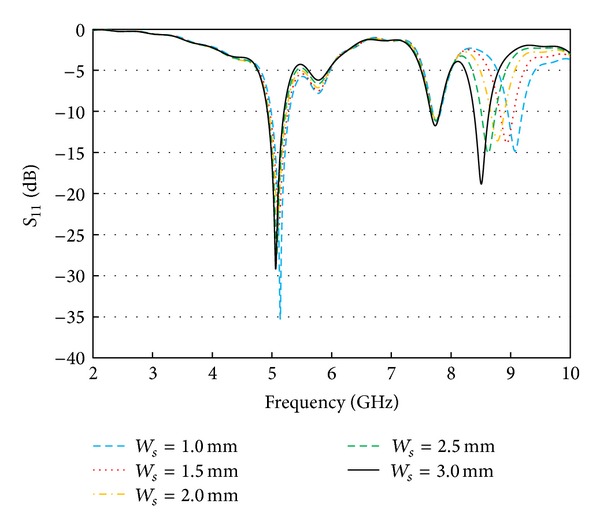
Effects of slot width (*Ws*).

**Figure 5:**
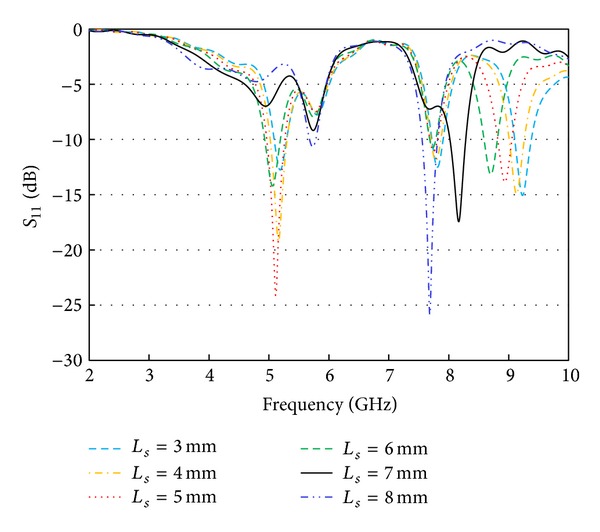
Effects of slot length (*Ls*).

The multiband nature of the proposed resonator is contributed by the embedded rectangular slots within the rectangular radiator. To understand their effects, the return loss profile is investigated with different slot length (*Ls*) and width (*Ws*). Either the length or the width is varied at once to realize the happenings. It is clear from Figure 4 that the width is not so dominant on the resonance at 5.2 GHz as it is on the resonance around 9 GHz. However, as shown in Figure 5, the slot length is found to be dominating and an essential parameter for obtaining multiple deep resonances. The slots are responsible for lengthening current path and the current distribution at different spots on the radiator and thus control the resonator characteristics.

### 3.3. Effects of the Slot Arrangements

**Figure 6:**
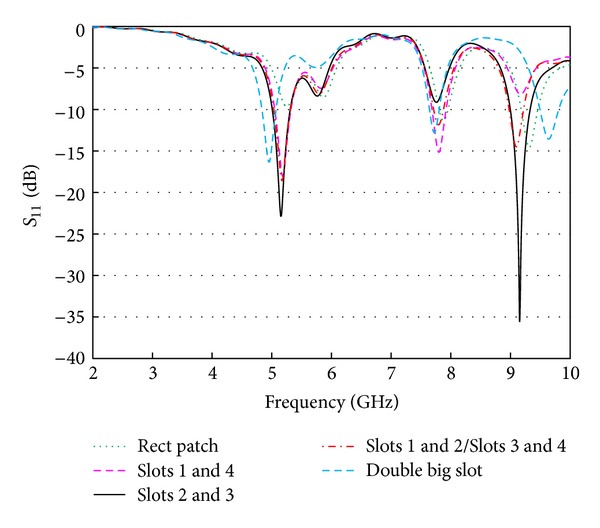
Effects of arrangements of different slots.

One of the main contributors in obtaining the multiband behaviors is the symmetrical slot arrangements.  Figure 6 shows the *S*_11_ characteristics for several slot arrangements. A rectangular radiator gives a resonance at 9 GHz with minimum return loss of −15 dB, while the radiator with two big horizontal slots shows a multiresonance nature, but the return loss level becomes poorer. Considering the slot effect, the big horizontal slots are half-sectioned and their positions are adjusted symmetrically on both sides of the feeding microstrip line. Thus, there are four symmetrical slots now on the resonator which are numbered as slot 1, slot 2, slot 3 and slot 4. As seen in the figure, either combination of slots 1 and 4, or slots 2 and 3 gives deep return losses while they still preserving the multi resonance nature. However, when slots placed on the left or right side (slots 1 and 2 or slots 3 and 4) of the feed line are active, the second and third resonances are affected and the return loss behavior is not as good as the previous arrangements. It is clear from the figure that the first resonance is almost stable at 5.2 GHz irrespective of the slot arrangement.

### 3.4. Effect of the Defected Ground Surface (DGS)

**Figure 7:**
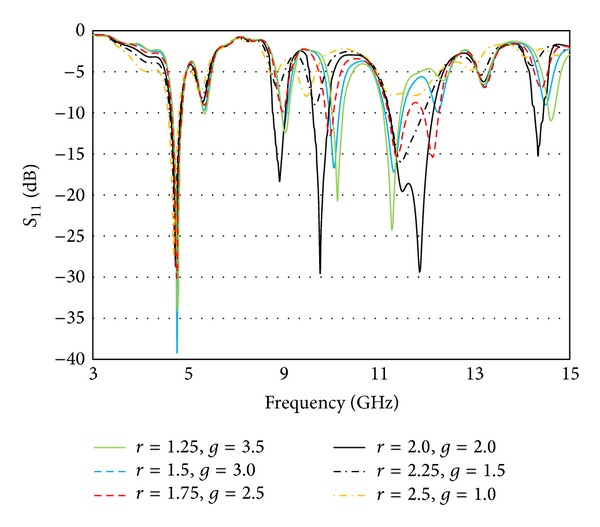
Variation of return loss characteristics with respect to the defect radius (*r*) and gap between adjacent defects (*g*).

Figure 7 is showing how the return loss characteristics are influenced by the defect radius (*r*) and defect gap (*g*). The insertion of ground slots created some sort of discontinuity, which caused the electric current launched by the primary radiator to reroute its path along the conducting surface of the ground. As a result, the electrical length of the ground is increased. With the strong coupling from the radiator, the ground slots cause a considerable impact on the input impedance. This positive impact includes the introduction of new resonance at around 14.5 GHz, which is also advantageous for a multiband resonator design.

## 4. Experimental Validation of the DGS Resonator Performances

### 4.1. Return Loss (*S*_11_) Characteristics

**Figure 8:**
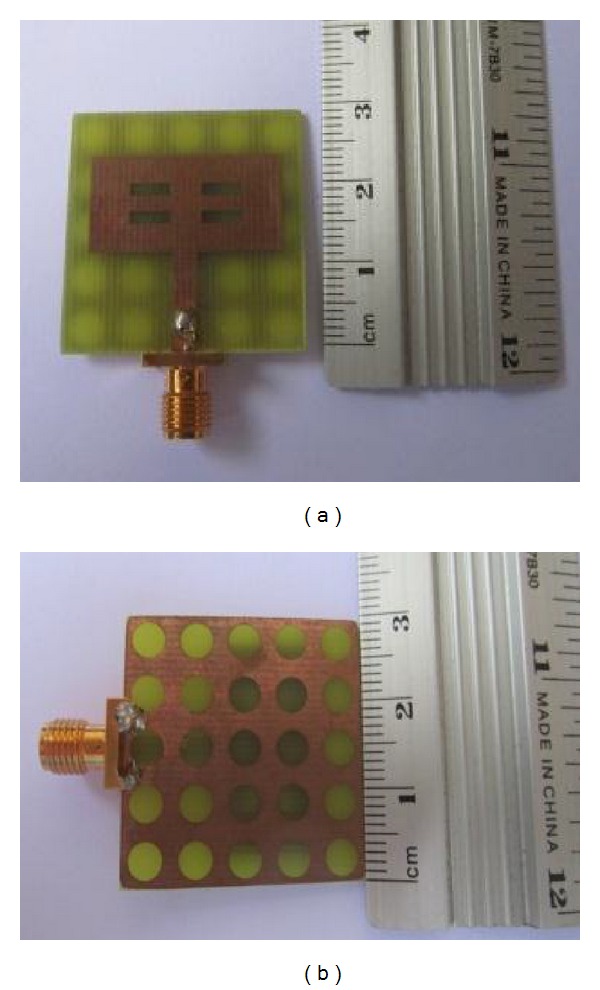
Prototyped DGS multiband resonator, (a) front side (b) back side.

**Figure 9:**
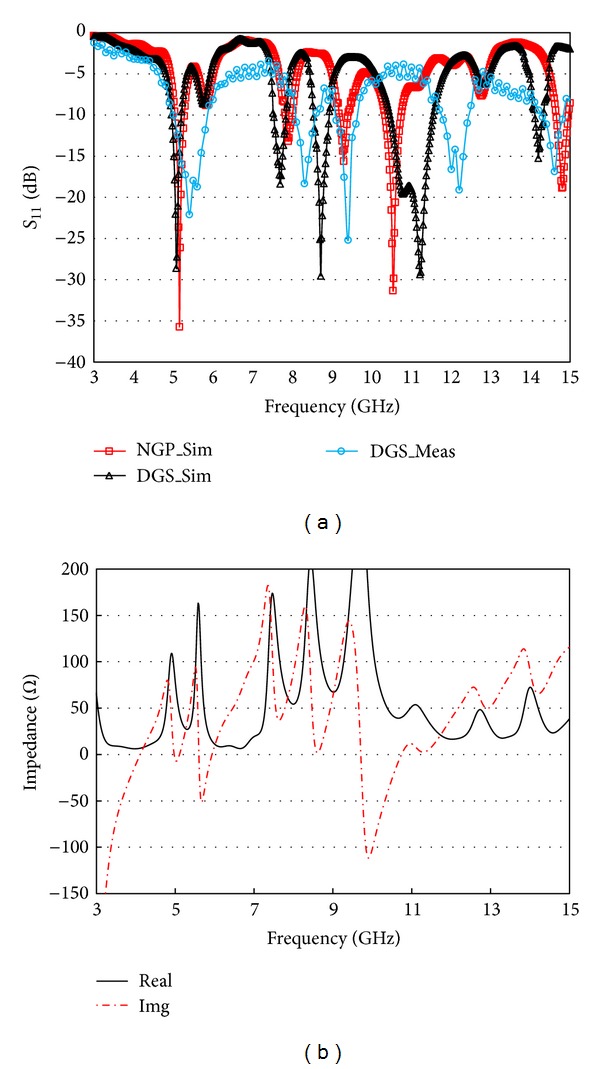
(a) Comparison of the simulated and measured return loss characteristics. (b) Impedance characteristics of the proposed DGS resonator.

With the consideration of the above parametric analyses, the resonator performances are investigated and compared with a normal ground plane (NGP) and a DGS (or defected ground plane, DGP). The simulated and measured *S*_11_ characteristics of the resonator with NGP and DGS are depicted in Figure 9(a).  Figure 8 is showing front and rear view of the prototyped resonator with a DGS. By introducing circular defects at a regular interval in the plain ground, comparatively better resonator performances are obtained.

The prototyped resonator's return loss characteristics are measured by using Agilent E8362C PNA network analyzer, and the radiation characteristics are investigated through the far field measurement system in an anechoic chamber. However, the impedance characteristics of the DGS resonator are depicted in Figure 9(b). The measured return loss behavior is very close to the simulated results except some resonance shifting. Differences between simulation and measurement can be attributed to the soldering roughness and fabrication imperfections.

### 4.2. Radiation Characteristics

**Figure 10:**
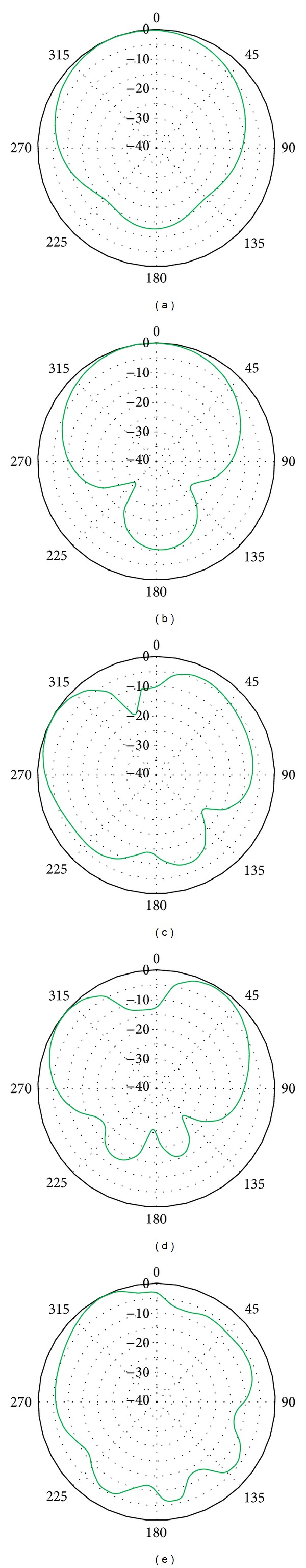
Simulated radiation characteristics of NGP resonator at (a) 5.2, (b) 7.9, (c) 9.3, (d) 10.5, and (e) 14.8 GHz.

**Figure 11:**
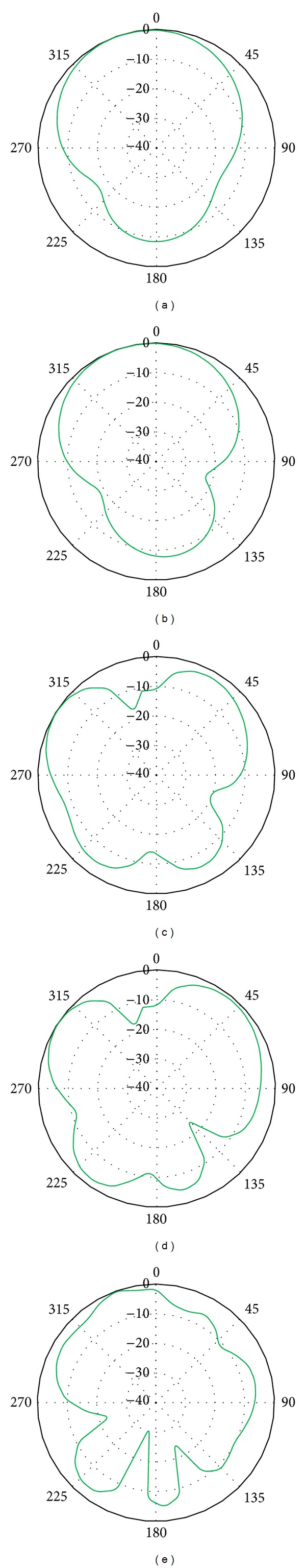
Simulated radiation characteristics of DGS resonator at (a) 5.11, (b) 7.7, (c) 8.7, (d) 11, and (e) 14.3 GHz.

The resonator radiation performances are assessed for all resonant frequencies with NGP and DGS, consequently.  Figure 10 shows the simulated radiation characteristics of the NGP resonator at the resonant points of 5.2, 7.9, 9.3, 10.5, and 14.8 GHz, respectively. Similarly, Figure 11 depicts the DGS resonator radiation patterns at the resonance of 5.11, 7.7, 8.7, 11, and 14.3 GHz, respectively.

The radiation patterns are normalized to the maximum value at the respective resonance frequencies for ease of understanding. As it can be seen, due to the defects in the ground the respective resonances shifted slightly from the NGP resonances, but overall radiation characteristics are improved as they become more directional and the back radiation is reduced.

**Figure 12:**
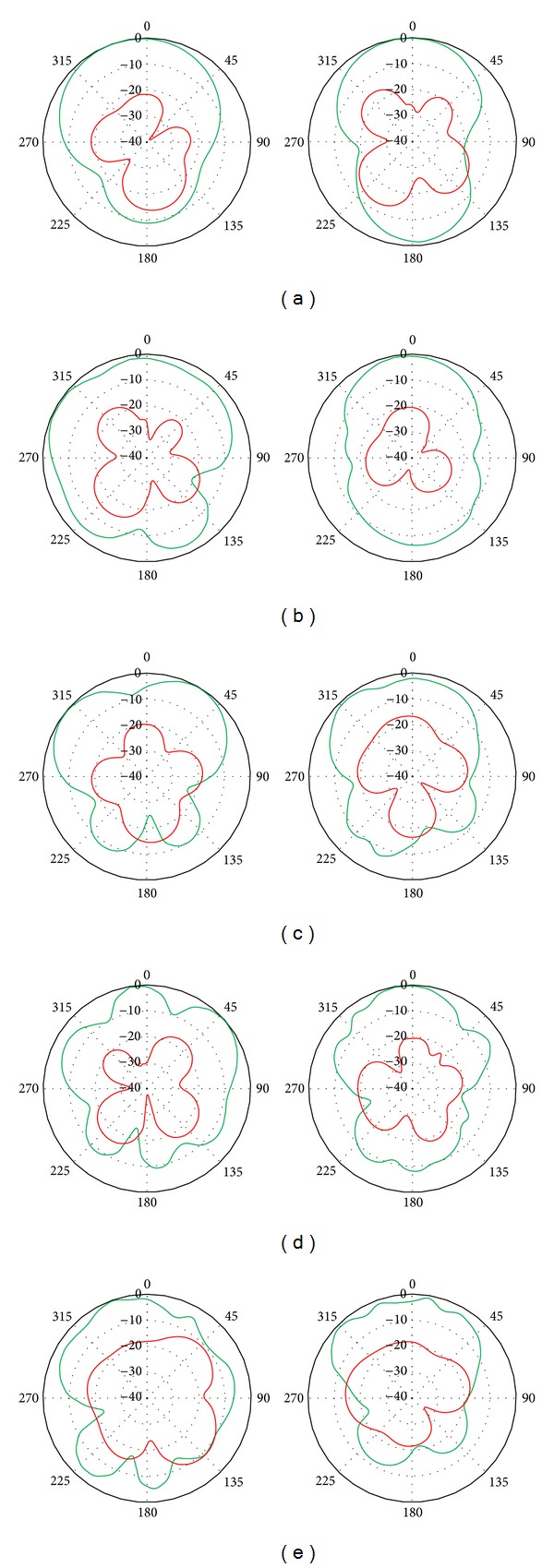
Measured radiation characteristics of the DGS resonator at (a) 5.5, (b) 8.4, (c) 9.5, (d) 12.2, and (e) 14.6 GHz. (Left side: E-plane, right side: H-plane.)

Measured radiation characteristics of the DGS (or DGP) resonator at the resonance frequency of 5.5, 8.4, 9.5, 12.2, and 14.6 GHz are shown in Figure 12. Despite having some unavoidable nulls at the measurement, the resonator radiates similarly to the simulations. The resonator radiations are found to be broader and more directional. The cross polarization level is also low, around −20 dB. The 3 dB angular beam widths are obtained as 86°, 53.7°, 43.9°, 42.1°, and 30.1°, respectively, at the resonances.

**Figure 13:**
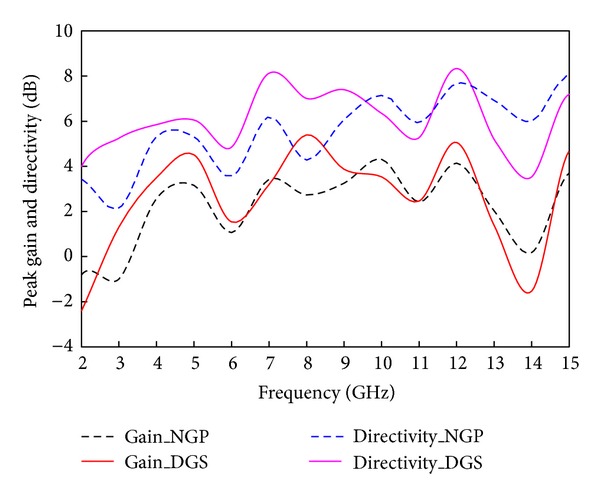
Maximum gain and directivity over the operating band for NGP and DGS.

The maximum gain and the directivity over the operating frequency band are plotted in Figure 13 for both NGP and DGS resonators. For the NGP resonator the gain peak found is 3.42 dBi at 7.5 GHz and average gain varies between 2.5 and 3 dBi over the frequency band of interest. In contrast, the DGS resonator gives a maximum gain of 6.2 dBi at 8.3 GHz, while the average gain is found to be around 5 dBi, and it is almost stable over the operating band. The periodical defects introduced into the conventional plain ground enhanced the resonator gain and the directivity by reducing the back radiations and side lobes, whilst they preserved the multiband nature of the proposed resonator. Furthermore, the resonances are not shifted much in the presence of the circular defects.  Table 1 concludes the multiband resonator performances in presence of the defected ground. Despite the multiresonant characteristics, this resonator still preserved high gain, high directivity, and good beam width. Moreover, the radiation main lobe stayed within 10° to 50°, which is advantageous as the resonator radiates in the front side mostly.

## 5. Conclusion

A multiband high gain microstrip resonator is presented which is realized with a rectangular radiator with four rectangular symmetrical slots inside and a defected ground plane. To obtain multiresonances with stable gain, the design parameters are chosen from a wide range of parametric studies. Then, a DGS resonator is fabricated on an epoxide woven glass fabric dielectric material and tested with a vector network analyzer and in an anechoic chamber for the practical validation. The resonator showed five resonances at 5.2, 8.3, 9.5, 12.2, and 14.6 GHz and achieved a peak gain of 5.85, 6.2, 3.9, 5.9, and 4.7 dBi, respectively. The main lobe magnitude and direction are 6.2, 5.9, 8.5, 8.6, and 4.7 dBi and 10°, 50°, 31°, 50°, and 10°, respectively, at 5.2, 8.3, 9.5 12.2, and 14.6 GHz resonant frequencies. Furthermore, the 3 dB angular beam widths are obtained as 86°, 53.7°, 43.9°, 42.1°, and 30.1°, respectively, at the resonances. It is observed that the DGS resonator has better performances than an NGP resonator in terms of resonator gain, directivity, and other radiation characteristics. Similar to the computed results by the simulator, measured performances are also found to be up to standard for practical application of the projected resonator.

## Figures and Tables

**Table 1:** The multiband DGS resonator performances.

Frequency (GHz)	Peak gain (dB)	Main lobe magnitude (dBi)	Main lobe direction	3-dB beam width
5.2	5.85	6.2	10°	86°
8.3	6.2	5.9	50°	53.7°
9.5	3.9	6.5	31°	43.9°
12.2	5.9	8.6	50°	42.1°
14.6	4.7	4.7	10°	30.1°
